# Age distribution and seasonality in acute eosinophilic pneumonia: analysis using a national inpatient database

**DOI:** 10.1186/s12890-019-0800-3

**Published:** 2019-02-12

**Authors:** Koshi Ota, Yusuke Sasabuchi, Hiroki Matsui, Taisuke Jo, Kiyohide Fushimi, Hideo Yasunaga

**Affiliations:** 1 Department of Clinical Epidemiology and Health Economics, School of Public Health, The University of Tokyo, 7-3-1, Hongo, Bunkyo-ku, Tokyo, 1130033 Japan; 2 Department of Emergency, Osaka Medical College, 2-7 Daigakumachi, Takatsuki, Osaka, 569-8686 Japan; 3 Data Science Center, Jichi Medical University, 3311-1 Yakushiji, Shimotsuke-shi, Tochigi-ken 329-0498 Japan; 4 Department of Health Services Research, Graduate School of Medicine, The University of Tokyo, 7-3-1, Hongo, Bunkyo-ku, Tokyo, 1130033 Japan; 5 Department of Health Policy and Informatics, Tokyo Medical and Dental University Graduate School, 1-5-45 Yushima, Bunkyo-ku, Tokyo, 113 - 8510 Japan

**Keywords:** Acute eosinophilic pneumonia (AEP), Age distribution, Bronchoscopy, Corticosteroid, Seasonality

## Abstract

**Background:**

Acute eosinophilic pneumonia (AEP) is a rare inflammatory lung disease. Previous studies have shown that most patients with AEP are aged 20 to 40 years, whereas several case studies have included older patients with AEP. These studies also suggested that AEP is more prevalent in summer, but they were limited due to their small sample sizes. We therefore investigated the age distribution and seasonality among patients with AEP using a national inpatient database.

**Methods:**

Using the Japanese Diagnosis Procedure Combination database, we identified patients with a recorded diagnosis of AEP from 1 July 2010 to 31 March 2015. We examined patient characteristics and clinical practices including age, sex, seasonal variation, length of stay, use of corticosteroids, use of mechanical ventilation, and in-hospital mortality.

**Results:**

During the 57-month study period, we identified 213 inpatients with AEP. The age distribution of AEP peaked twice: at 15 to 24 years and 65 to 79 years. The proportion of patients with AEP was highest in summer for those aged < 40 years, whereas it was distributed evenly throughout the year for those aged ≥ 40 years. The interval from hospital admission to corticosteroid administration and the duration of corticosteroid use were significantly longer in the older than younger age group.

**Conclusions:**

The age distribution of patients with AEP was bimodal, and seasonality was undetected in older patients. Older patients may be more likely to have delayed and prolonged treatment.

## Background

Acute eosinophilic pneumonia (AEP) is a rare disease that was originally reported by Badesch et al. [1] and Allen et al. [2] in 1989. Patients with AEP frequently show hypoxaemic respiratory dysfunction and often require mechanical ventilation [3, 4]. Previous studies have indicated that patients with AEP have a rapid response to corticosteroid treatment [1, 2, 4, 5] and low mortality. However, a delay in diagnosis and treatment may result in increased mortality [6–9].

Most patients with AEP in previous studies were aged 20 to 40 years [3, 5, 8, 10–14], but several case reports and case series have included older patients [15–18]. However, these previous studies were limited due to their small number of patients. Studies regarding the seasonality of AEP occurrence included only young healthy military personnel [5, 8, 10, 11].

We therefore investigated the age distribution and seasonality among patients with AEP who required hospitalization using a nationwide inpatient database in Japan. We also examined clinical practices for patients with AEP and compared them between young and older patients.

## Methods

### Data source

For this study, we used the Diagnosis Procedure Combination database from 1 July 2010 to 31 March 2015. All 82 academic hospitals in Japan are obliged to participate in the database, while participation by community hospitals is voluntary. The database includes administrative claims data and some clinical data for all inpatients. The database contains the following items: unique hospital identifiers, patient age and sex, type of procedures, length of stay, and diagnoses and comorbidities recorded in Japanese text and International Classification of Diseases 10th Revision (ICD-10) codes. Dates of procedures performed and drugs prescribed are also recorded. To optimize the accuracy of diagnoses, attending physicians are required to record the diagnoses with reference to medical charts.

Given the anonymous nature of the data, informed consent was not required for this study. The research was approved by the Institutional Review Board at The University of Tokyo.

### Patient selection

**Fig. 1:**
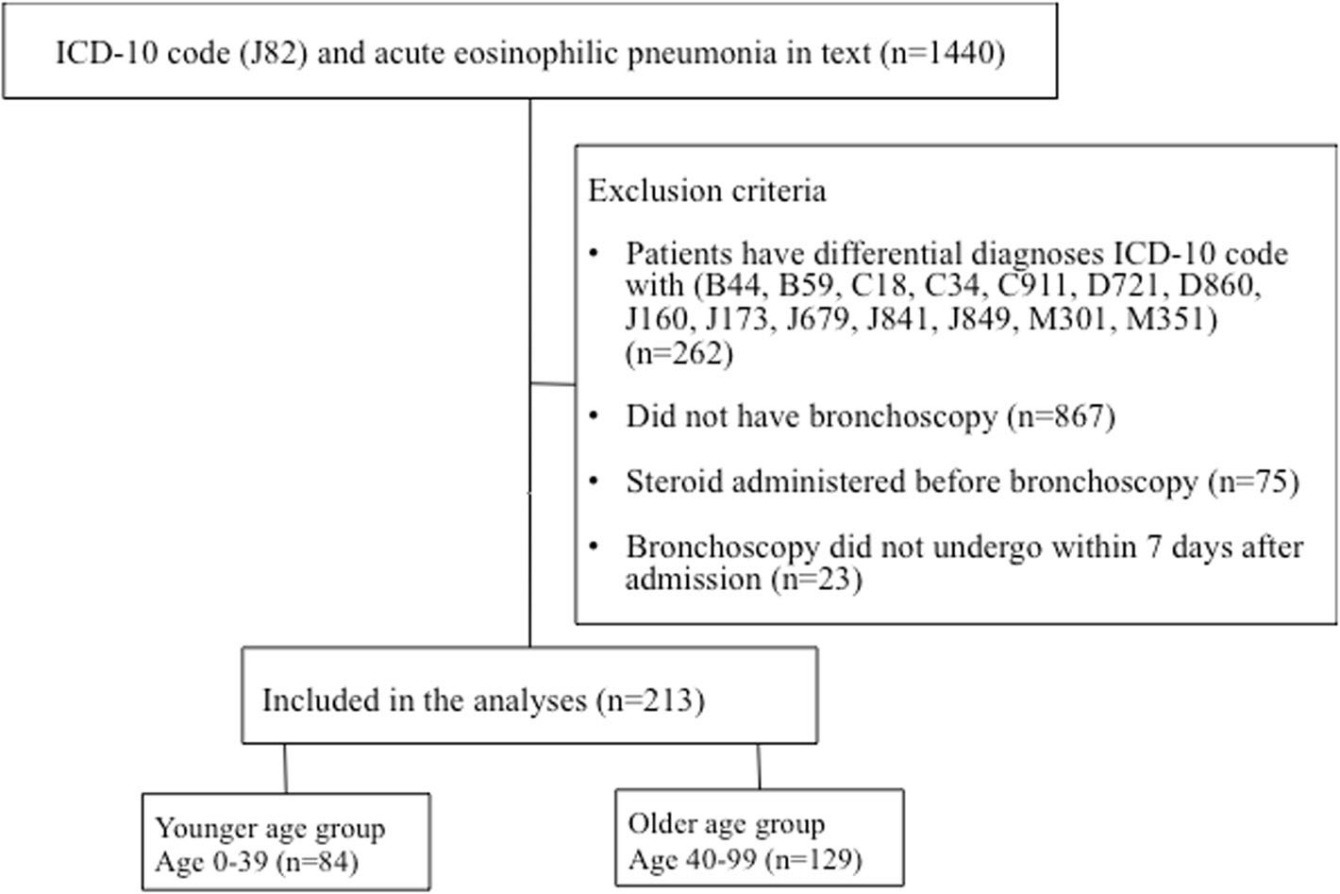
Inclusion and exclusion criteria

We identified patients with an ICD-10 code of J82 (pulmonary eosinophilia) and whose diagnosis in Japanese text was ‘acute eosinophilic pneumonia’. Among them, we excluded patients who also had any of the following ICD-10 codes: aspergillosis (B44), pneumocystosis (B59), pneumonia in parasitic diseases (J173), lung cancer (C34), chronic lymphocytic leukaemia (C911), eosinophilia (D721), sarcoidosis of the lung (D860), chlamydial pneumonia (J160), hypersensitivity pneumonitis (J679), pulmonary fibrosis (J841), interstitial pulmonary disease unspecified (J849), eosinophilic granulomatosis with polyangiitis (Churg–Strauss syndrome) (M301), other overlapping syndromes (M351), and colon cancer (C18). Because AEP is confirmed with bronchoscopy and corticosteroid therapy is generally provided after bronchoscopy, we excluded patients who did not undergo bronchoscopy within 7 days after admission and those who received corticosteroids before bronchoscopy to improve the specificity of the diagnosis of AEP [2, 3]. (Fig. 1).

### Patient backgrounds

Patient background data included age, sex, smoking status, Charlson comorbidity index (CCI) at admission, and season of admission. The CCI was calculated as a weighted score of specific comorbid diseases based on the ICD-10 codes [19]. Seasons were defined as the following 3-month periods: spring as March to May, summer as June to August, fall as September to November, and winter as December to February [20].

### Clinical practice

Data on patients’ clinical practices included length of stay after bronchoscopy, interval (days) from admission to bronchoscopy, interval (days) from admission to corticosteroid administration, duration of corticosteroid therapy, use of mechanical ventilation, and in-hospital death.

### Statistical analyses

According to previous reviews of AEP [4], patients aged < 40 years were categorized as the younger age group, and those aged ≥ 40 years were categorized as the older age group. Patient backgrounds and clinical practices were compared between the younger and older age groups. Categorical variables are presented as numbers with percentages and were compared using the chi-squared test. Continuous variables are presented as median and interquartile range and were compared using the Mann–Whitney U test.

For each group (younger and older patients with AEP), we described the seasonality of AEP admissions and compared the proportions of patients with AEP among the four seasons using chi-squared tests.

A *P* value of < 0.05 was considered statistically significant. All statistical analyses were performed using IBM SPSS version 23 (IBM Corp., Armonk, NY, USA).

## Results

During the 57-month study period, we identified 213 eligible patients. Figure 1 shows the algorithm for patient selection.

**Fig. 2:**
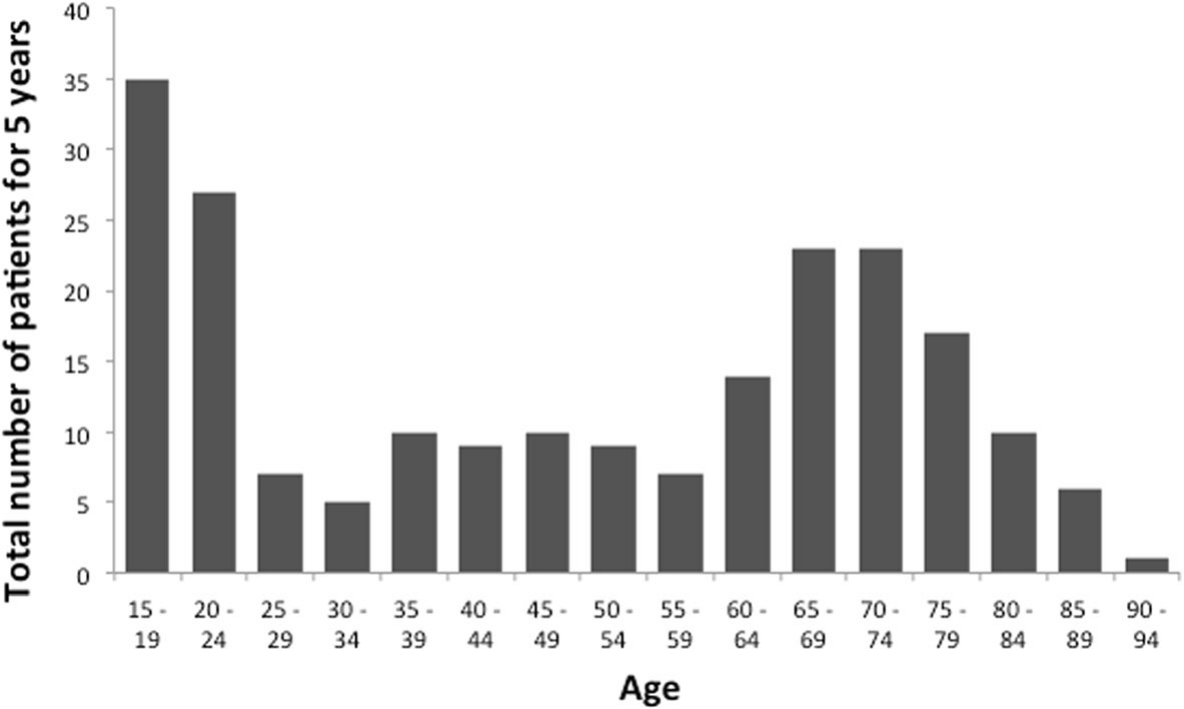
Age distribution of patients with acute eosinophilic pneumonia

Of these, 84 patients were in the younger age group and 129 patients were in the older age group. The age distribution of all patients with AEP is shown in Fig. 2. There was a bimodal distribution peaking around age 15 to 24 and 65 to 79 years.

Table 1 shows the patient characteristics of the two age groups. Although not significant, the proportion of males was higher in the younger than older age group. The smoking status was not significantly different between the two groups. The older age group was significantly more likely to have a higher CCI. With respect to seasonality, about one-half of AEP admissions in the younger age group occurred in summer. Seasonality was significantly different between the two groups.

**Table 1:** The prevalence of HPV infection in all the specimens

	< 40 years	≥ 40 years	
	(*n* = 84)	(*n* = 129)	P
Male	55 (65.5)	68 (52.7)	0.065
Smoking status			
Never-smoker	39 (46.4)	75 (58.1)	0.113
Current or past smoker	31 (36.9)	43 (33.3)	
Missing data	14 (16.7)	11 (8.5)	
Charlson comorbidity index			
0	69 (82.1)	74 (57.4)	0.002
1	12 (14.3)	37 (28.7)	
2	2 (2.4)	12 (9.3)	
3	1 (1.2)	6 (4.7)	
Season			
Spring	13 (15.5)	25 (19.4)	0.001
Summer	40 (47.6)	27 (20.9)	
Fall	13 (15.5)	35 (27.1)	
Winter	18 (21.4)	42 (32.6)	

Data are presented as n (%)

**Fig. 3:**
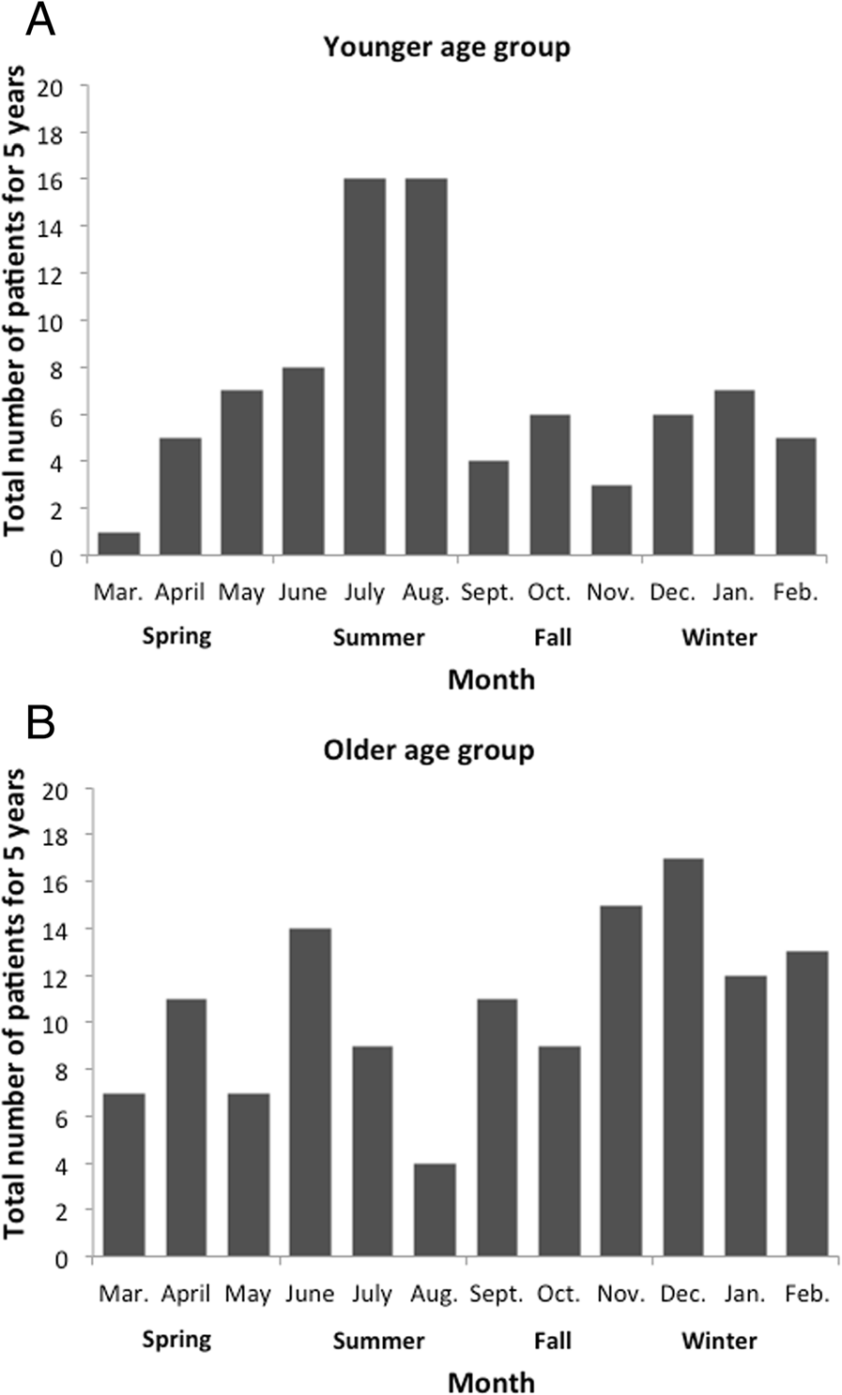
Seasonality of acute eosinophilic pneumonia in patients aged (**a**) < 40 years and (**b**) ≥ 40 years

Figure 3 shows the seasonal distribution of hospitalized patients with AEP in the two age groups. In the younger age group, a distinct peak was observed in summer (July–August). Chi-squared tests showed a significant difference in the proportions of AEP admissions among the four seasons in the younger age group (*P* < 0.001), but not in the older age group (*P* = 0.129).

Table 2 compares the clinical practices between the younger and older age groups. The older age group showed a significantly longer length of stay after bronchoscopy, interval from admission to corticosteroid administration, and duration of corticosteroid administration compared with the younger age group. There was no significant difference in the use of mechanical ventilation or in-hospital death between the two groups.

**Table 2:** Clinical courses of patients with acute eosinophilic pneumonia in the younger and older age groups

	< 40 years	≥ 40 years	
	(*n* = 84)	(*n* = 129)	P
Length of stay after bronchoscopy (days)	8 (5–11)	16 (9–26)	< 0.001
Days from admission to bronchoscopy	2 (1–3)	2 (1–4)	0.006
Days from admission to corticosteroid administration	2 (1–3)	3 (2–5)	< 0.001
Duration of corticosteroid use, days	7 (4–11)	15 (9–25)	< 0.001
Mechanical ventilation	5 (6.0)	11 (8.5)	0.486
Death	0 (0.0)	4 (3.1)	0.103

Data are presented as median (interquartile range) or n (%)

## Discussion

Using a national inpatient database in Japan, we identified two peaks in the age distribution of patients with AEP. The younger patients were hospitalized more frequently in summer, whereas there was no significant seasonal variation in hospitalization for AEP in the older patients.

Previous studies have suggested that AEP mainly occurs in younger patients [3, 5, 8, 10–14]. In contrast, the population in the present study included patients of all ages from the national database. Notably, most patients with AEP in our study were aged ≥ 40. In fact, the proportions of older patients in two previous small case series and one clinical study were comparable with the proportion in our study [18, 21, 22].

The sex ratio of patients with AEP was inconsistent among previous studies and understandably male-dominant in the military cohort [5, 8, 10, 11]. In several case series [3, 16, 23, 24], the occurrence of AEP was similar between male and female patients. In the present study, AEP was more common in men in the younger age group, whereas the occurrence of AEP was similar between men and women in the older age group. The reason for this remains unclear, however toxin inhalations, infections, and medications may be able to explain this [22, 25].

As in previous reports [8, 11], the occurrence of AEP in the younger age group was dominant in summer, whereas that in the older age group did not show such a trend. This may suggest a difference in causal factors of AEP between younger and older patients. Although the present study cannot clarify the causes of AEP, we speculate that several reported factors, including susceptibility to medication [22, 25], air pollution [26, 27] and viral infection [28, 29], may have differed between the younger and older patients. This might have caused the difference in their seasonal variation.

The length of stay after bronchoscopy, interval from admission to corticosteroid administration, and duration of corticosteroid therapy were significantly different between the younger and older age groups. This may suggest that older patients are more likely to have delayed and prolonged treatment.

Our study has several limitations. First, data on laboratory testing, imaging, and histopathology were not available in the database. Second, pre-admission data were also unavailable. Third, we may have overlooked some older patients with AEP, possibly because physicians may have hesitated to perform bronchoscopy in such patients.

## Conclusions

In conclusion, our study showed that patients hospitalized with AEP had a bimodal age distribution. Younger patients were more prevalent in summer, but AEP in older patients did not show seasonality.

## Abbreviations

AEP: Acute eosinophilic pneumonia
CCI: Charlson comorbidity index
ICD-10: International Classification of Diseases 10th Revision
